# A resting-state fMRI cross-sectional study of cardiorespiratory fitness decline after stroke

**DOI:** 10.3389/fneur.2025.1465467

**Published:** 2025-01-28

**Authors:** Qingming Qu, Kexu Zhang, Hewei Wang, Jie Zhu, Yingnan Lin, Jie Jia

**Affiliations:** ^1^Department of Rehabilitation Medicine, Affiliated Hospital of Nantong University, Nantong, Jiangsu, China; ^2^School of Biomedical Engineering, Shanghai Jiao Tong University, Shanghai, China; ^3^Department of Rehabilitation Medicine, Huashan Hospital, Fudan University, Shanghai, China; ^4^National Center for Neurological Disorders, Shanghai, China; ^5^Department of General Medicine, Huashan Hospital, Fudan University, Shanghai, China; ^6^National Clinical Research Center for Aging and Medicine, Huashan Hospital, Fudan University, Shanghai, China

**Keywords:** cardiorespiratory fitness, resting-state fMRI, amplitude of low-frequency fluctuations, functional connectivity, brain network

## Abstract

**Objective:**

The present study aimed to investigate alterations in neural activity and reorganization of functional networks within critical brain regions associated with reduced cardiorespiratory fitness (CRF) in stroke patients. By employing resting-state functional magnetic resonance imaging (fMRI), we sought to identify specific brain areas that may be implicated in CRF decline among this patient population.

**Methods:**

A total of 22 patients with stroke and 15 healthy subjects matched for age, gender, and body mass index were recruited. Rehabilitation assessments included peak oxygen uptake (VO_2_peak), peak work-rate, 10-meter walk test (10mWT), five times sit-to-stand test (FTSST), and 6-min walking distance (6MWD). Resting-state fMRI data were collected for the two groups, and correlation between changes in the amplitude of low-frequency fluctuations (ALFF) and CRF was analyzed to detect brain regions related to CRF and local neural activity in patients with stroke. On the basis of ALFF analysis, brain network analysis was performed, and the CRF-related brain regions in patients with stroke were selected as seed points. Functional connectivity (FC) analysis was the used to identify brain regions and networks potentially associated with CRF in patients with stroke.

**Results:**

Patients with stroke exhibited significantly lower VO_2_peak, peak work-rate, 10mWT, and 6MWD compared to healthy controls (*p* < 0.001). FTSST was significantly higher in patients with stroke than healthy controls (*p* < 0.001). ALFF analysis identified CRF-related brain regions in patients with stroke, including the ipsilesional superior temporal gyrus (*r* = 0.56947, *p* = 0.00036), middle frontal gyrus (*r* = 0.62446, *p* = 0.00006), and precentral gyrus (*r* = 0.56866, *p* = 0.00036). FC analysis revealed that the functional connectivity of brain regions related to CRF in patients with stroke involved the ipsilesional M1 to ipsilesional precentral gyrus and contralesional postcentral gyrus, and the correlation coefficients were *r* = 0.54802 (*p* = 0.00065) and *r* = 0.49511 (*p* = 0.0025), respectively. The correlation coefficients of ipsilesional middle frontal gyrus to contralesional middle frontal gyrus, angular gyrus and ipsilesional superior frontal gyrus were *r* = 0.58617 (*p* = 0.00022), *r* = 0.57735 (*p* = 0.00028), and *r* = −0.65229 (*p* = 0.00002), respectively.

**Conclusion:**

This study observed that CRF levels were lower in stroke patients compared to those in healthy individuals. Resting fMRI analysis was applied to identify CRF-related brain regions (ipsilesional superior temporal, middle frontal, precentral gyri) and networks in patients with stroke.

**Clinical trial registration:**

https://www.chictr.org.cn/showproj.html?proj=151095.

## 1 Introduction

In China, a country with the largest stroke burden in the world, stroke (including ischemic stroke and hemorrhagic stroke) is the leading cause of mortality (1), and it is the third most common cause of disability-adjusted life years lost worldwide (2). It is well known that stroke is associated with many long-term complications, such as impairments in motor, language, and cognitive functions (3). Additionally, stroke can lead to a decline in cardiorespiratory fitness (CRF). CRF is the ability of the body to transport and use oxygen, usually expressed as maximal oxygen uptake (VO_2_max) or peak oxygen uptake (VO_2_peak) (4) and is considered a core component of health-related fitness (5). A low or unhealthy CRF is an independent and strong predictor of cardiovascular disease and adult all-cause mortality (6). The CRF of the stroke population, at an average level of 15.78 mL/kg/min (7), is approximately 53% of that of age-matched healthy populations (8). This CRF level is predictive of an inability to maintain daily activities and may be associated with a range of adverse outcomes, including frailty, reduced physical performance, an increased risk of cardiovascular events, and an increased rate of recurrent stroke (9).

Neuroplasticity, which can be defined as the ability of the brain to change its structure or function after exposure to new stimuli or environments (10), plays an important role in the recovery of patients with stroke (11). To the best of our knowledge, very little research has focused on whether CRF affects the recovery of stroke through neuroplasticity, and there have been no firm conclusions. Functional magnetic resonance imaging (fMRI), which can reflect the evolution of cortical functional remolding (12), has evolved rapidly in recent times, and resting state (rs)-fMRI especially has been applied to the study of many neurological disorders (13). Therefore, we used rs-fMRI to explore the brain regions associated with CRF to better understand the mechanisms affecting functional recovery in patients with stroke.

## 2 Methods

### 2.1 Study population

**Figure 1:**
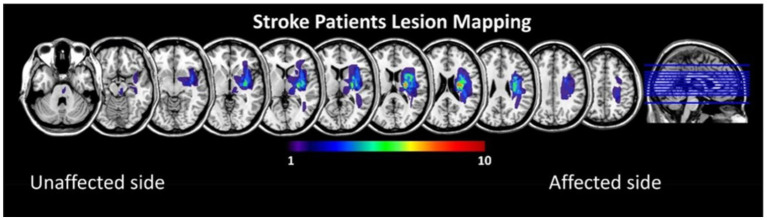
Lesion overlay maps for the stroke patients, all left lesions were flipped to the right side.

All stroke subjects were hospitalized in the department of rehabilitation medicine of Huashan Hospital Jing'an Branch of Fudan University from January to October 2022. A total of 58 patients with stroke with limb motor dysfunction were screened, and 22 patients (13 males and 9 females, with an average age of 57.2 ± 11.8 years) completed the trial and were ultimately included in the study. General patient information is shown in Table 1, and the superposition of lesions in the stroke subjects is shown in Figure 1. At the same time, 15 age-and sex-matched healthy volunteers (6 males and 9 females, mean age 57.3 ± 13.7 years) were recruited as healthy controls in Shanghai, and their general information is shown in Table 2.

**Table 1:** Demographic and clinical information for stroke patients.

Number	Sex	Age	Ischemia (I)/Hemorrhage (H)	Lesion	Days after stroke onset	Side of lesion, left/right	Hemiplegic side
S1	M	45	H	BG	365	R	L
S2	M	58	I	BG	90	R	L
S3	F	53	I	BG	360	R	L
S4	M	68	I	CR	361	L	R
S5	M	38	I	BG	150	R	L
S6	M	53	I	Pon	120	L	R
S7	M	51	H	BG	120	R	L
S8	F	73	I	BG	30	L	R
S9	M	65	I	BG	60	R	L
S10	F	22	I	BG, IC	30	R	L
S11	F	65	I	CR	362	R	L
S12	M	52	I	CR	32	L	R
S13	F	57	H	BG	400	R	L
S14	F	72	I	Pon	60	L	R
S15	F	58	I	BG	181	L	R
S16	F	66	I	BG, IC	14	L	R
S17	M	52	H	Tha	35	L	R
S18	M	55	I	Pon	397	L	R
S19	M	72	I	BG	99	L	R
S20	F	71	I	BG	21	R	L
S21	M	70	I	BG	210	L	R
S22	M	49	I	BG	389	L	R

S, stroke; F, female; M, male; I, ischemia; H, hemorrhage; L, left; R, right; BG, basal ganglia; CO, centrum ovale; CR, corona radiate; Tha, thalamus; IC, internal capsule.

**Table 2:** Demographic and clinical information for the participants.

	S (*n* = 22)	HC (*n* = 15)	Test statistics	*P*
	*M* (Q1–Q3)	*M* (Q1–Q3)		
Sex (M/F)	13/9	6/9	/	0.325
Age	57 (52, 68)	65 (48, 67)	*Z* = −1.567	0.117
BMI (kg/m^2^)	24.0 (22.8, 26.0)	24.3 (20., 25.9)	*Z* = −0.537	0.592
Weight (kg)	63.5 (56.5, 72.5)	62.5 (51, 69)	*Z* = −0.868	0.386
FTSST (s)	13.0 (11.4, 15.0)	7.7 (6.4, 10.0)	Z = −21.791	0.000
VO_2_ (ml/min)	895.7 (662.1, 1276.2)	1269.0 (1049.4, 1431.3)	*Z* = −2.351	0.019
VO_2peak_ (ml/kg/min)	14.3 (10.7, 18.7)	19.6 (16.3, 25.3)	*Z* = −15.539	0.000
Work-rate_peak_ (W)	75 (60, 90)	80 (70, 100)	*Z* = −7.218	0.000
6MWD (m)	190 (128, 330)	535 (493, 553)	*Z* = −24.835	0.000
10mWT (m/s)	1.0 (0.5, 1.3)	1.8 (1.6, 2.0)	*Z* = −19.934	0.000

S, stroke; F, female; M, male; HC, healthy controls.

All stroke subjects met the diagnostic criteria of “Diagnostic points for Major Cerebrovascular Diseases in China 2019” and were diagnosed as having stroke by computed tomography (CT) or magnetic resonance imaging (MRI). Inclusion criteria: (1) first onset, mainly subcortical injury; (2) 18 years ≤ age ≤ 80 years; (3) stable vital signs, clear consciousness, no obvious visual or hearing impairments, able to understand and execute commands; (4) unilateral limb movement disorder, Brunnstrom lower limb motor function grade IV; (5) mRS ≤ 3; (6) tolerance of cardiopulmonary exercise testing (CPET); (7) informed consent was obtained from patients or their immediate family members. Exclusion criteria: (1) history of heart, lung, or other diseases that seriously affect cardiopulmonary function; (2) severe spasticity (modified Ashworth Spasticity scale >2) or limited range of motion of the hemiplegic lower limb; (3) previous muscle, bone, and other serious diseases affecting power cycling pedaling; (4) patients with severe dementia or mental disorders; (5) patients with a cardiac pacemaker, metal joint replacement, claustrophobia, or other contraindications to MRI examination.

The inclusion criteria for the healthy subjects were as follows: (1) matched with the age, gender, and BMI of stroke subjects; (2) no major underlying diseases; (3) tolerance of MRI examination; (4) able to complete CPET; (5) fully understood the study and signed the informed consent form. Exclusion criteria were (1) history of stroke and (2) participants in other clinical trials.

This study was reviewed and approved by the Ethics Review Committee of Huashan Hospital, Fudan University. The Chinese clinical trial registry number is ChiCTR2200056043, and the registration time was January 31, 2022. All patients signed the written informed consent before enrolment, and the study was conducted in accordance with the Declaration of Helsinki.

### 2.2 Clinical outcome measurements and imaging data acquisition

Patients with stroke and healthy controls participated in 6-min walking distance (6MWD), 10-meter walk test (10mWT), five times stand-to-sit test (FTSST), VO_2_peak, and peak work-rate assessments. VO_2_peak and peak work-rate were assessed using CPET. Images of all subjects were collected with a 3.0-T MRI scanner (GE MR750, United States). MRI scanning included a T1-weighted scan, a T2-weighted scan, and a resting functional scan. Detailed information on the scanning and image preprocessing is included in the Supplementary material.

### 2.3 CPET

The study employed standardized CPET measuring equipment, utilizing a cycle ergometer, spirometry analysis, continuous gas exchange analysis (measuring oxygen consumption VO_2_peak, ml/kg/min), and electrocardiogram monitoring for heart rate analysis to assess baseline fitness levels (PowerCube Ergo, Schiller, Switzerland). CPET was conducted under the supervision of a medical doctor with expertise in cardiopulmonary resuscitation.

The CPET involved a ramp protocol that incorporated a multistage incremental step test. Following a 3-min warm-up at a power output of 0 W and a pedal cadence of 50 revolutions per minute, the workload was increased by 15–20 W every 2 min until participants reached a state of self-perceived exhaustion, characterized by an inability to maintain the prescribed pedal cadence, cardiovascular or pulmonary distress, or fatigue. Objective exhaustion was verified when the respiratory exchange rate exceeded 1.05. Following peak exercise, a 3-min recovery phase was administered to all participants. The calculation of oxygen consumption at the anaerobic threshold (ml/kg/min) was carried out using the modified V-slope method, as outlined by Wasserman (14). The definition of VO_2_peak was the highest level of oxygen consumption attained during the exercise test. It should be noted that this definition represents the maximum value, rather than an average of multiple breaths (15).

### 2.4 ALFF and FC analyses

The DPARSF toolbox was utilized to conduct the amplitude of low-frequency fluctuations (ALFF) analysis (16). The preprocessed image’s time series for each voxel underwent fast Fourier transformation to convert it into the frequency domain. The ALFF value was calculated for each voxel in the slow-5 band by dividing the power in the 0.01–0.027 Hz frequency range by the power in the full frequency range (0–0.25 Hz). The resulting ALFF maps were transformed into Z-score maps using Fisher’s Z-transformation.

Functional connectivity (FC) analysis was conducted using the CONN toolbox (17). The preprocessed images underwent band-pass filtering within the frequency range of 0.01–0.08 Hz. The voxel exhibiting the lowest *p*-value in the ALFF analysis (with detailed coordinates listed in the results) was designated as the center of a spherical seed (radius: 8 mm). The Pearson correlation coefficient was calculated for the relationship between the time series of each voxel and the seed to determine the FC.

### 2.5 Statistical analysis

We used SPSS (IBM SPSS 25.0, SPSS Inc.) to perform statistical analysis. The normality of function scores was assessed using the Shapiro–Wilk test, and measurement data are presented as mean ± SD for normally distributed data and median (Q1, Q3) for non-normally distributed data. The independent samples *t*-test or non-parametric Mann–Whitney *U* test was employed to determine statistically significant differences between groups. Categorical data were analyzed using the chi-square test, and the Fisher’s exact test probability method was utilized. Pearson’s correlation was utilized for correlation analysis. A *p*-value of ≤0.05 was deemed significant.

The SPM software was applied to analyze the resting fMRI data. In an effort to mitigate the occurrence of multiple comparison errors, threshold-free cluster enhancement (TFCE corrected *p* < 0.01, *H* = 2, *E* = 0.5, number of permutations = 5,000) with a cerebral gray matter mask was implemented. A statistically significant difference was established through line correction, with a *p*-value of less than 0.05. The results were presented using Xjview software. To further validate the relationship between the observed changes in brain activity and the patient’s CRF, a Pearson correlation assay was employed to calculate the correlation between ALFF, FC, and VO_2_peak change.

## 3 Results

### 3.1 Behavior

**Figure 2:**
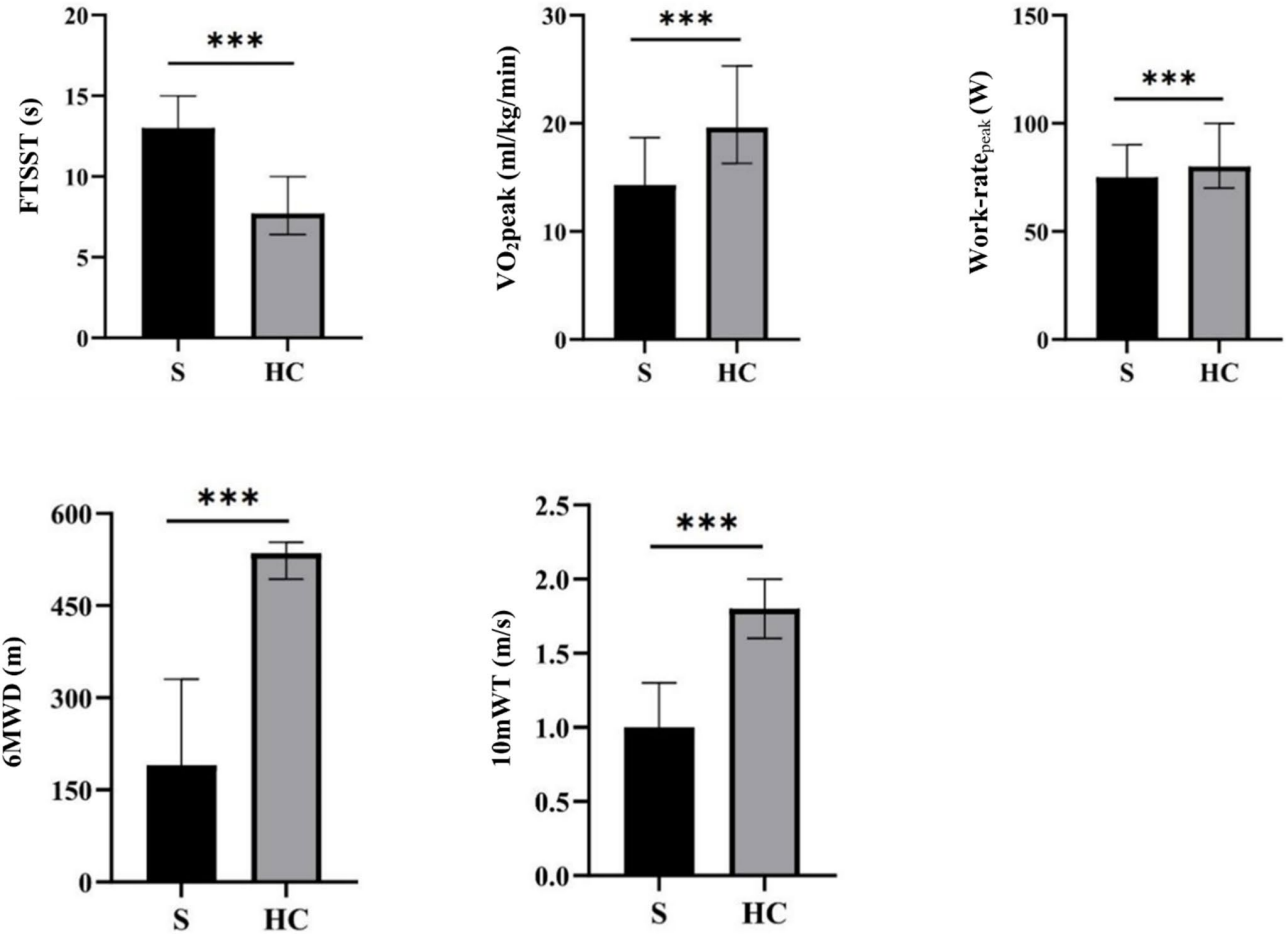
An analysis of behavioral data for stroke patients and healthy controls. *p < 0.05, **p < 0.01, ***p < 0.001. FTSST, five times sit-to-stand test; VO_2_peak, peak oxygen uptake; 6MWD, 6-min walking distance; 10mWT, 10-meter walk test.

Statistical analysis revealed that patients with stroke exhibited significant differences in various physical performance measures compared to healthy controls. Specifically, patients with stroke demonstrated increased performance in the FTSST, while exhibiting decreased CRF, peak work-rate, 6MWD, and 10mWT performance (Table 2 and Figure 2).

### 3.2 ALFF

**Figure 3:**
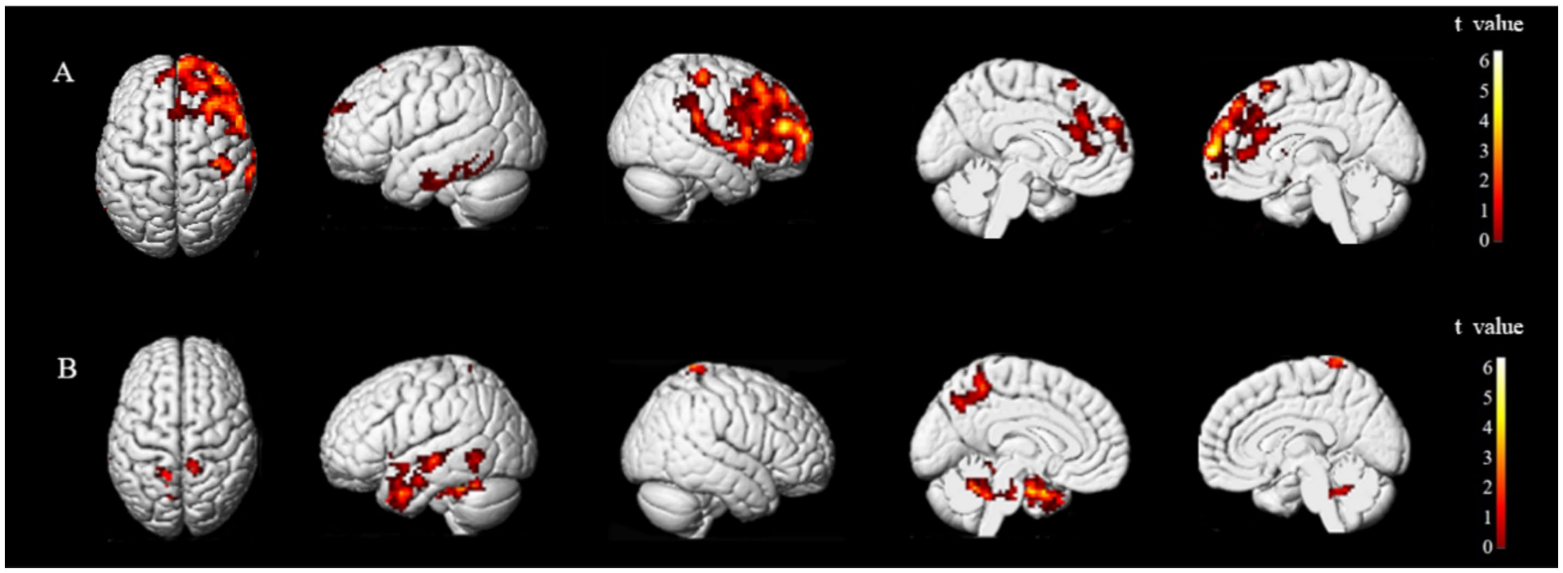
Brain regions with differences in ALFF between two groups. ALFF values were subjected to TFCE correction with a significance level of *p* < 0.005 and a cluster threshold of >30. Group A and Group B represent brain regions of stroke patients with reduced and increased ALFF values, respectively. The *t*-value is depicted in the color bar chart.

The brain regions exhibiting statistically significant differences in ALFF between the two groups were the middle frontal gyrus (MFG) and precentral gyrus (preCG), and superior temporal gyrus (STG) on the ipsilesional side, and the superior parietal lobule (SPL) on the bilateral side. Notably, patients with stroke demonstrated decreased ALFF value for their MFG and anterior central gyrus on the ipsilesional side, while an increased ALFF value was observed for their ipsilesional STG and bilateral SPL (Figure 3).

**Figure 4:**
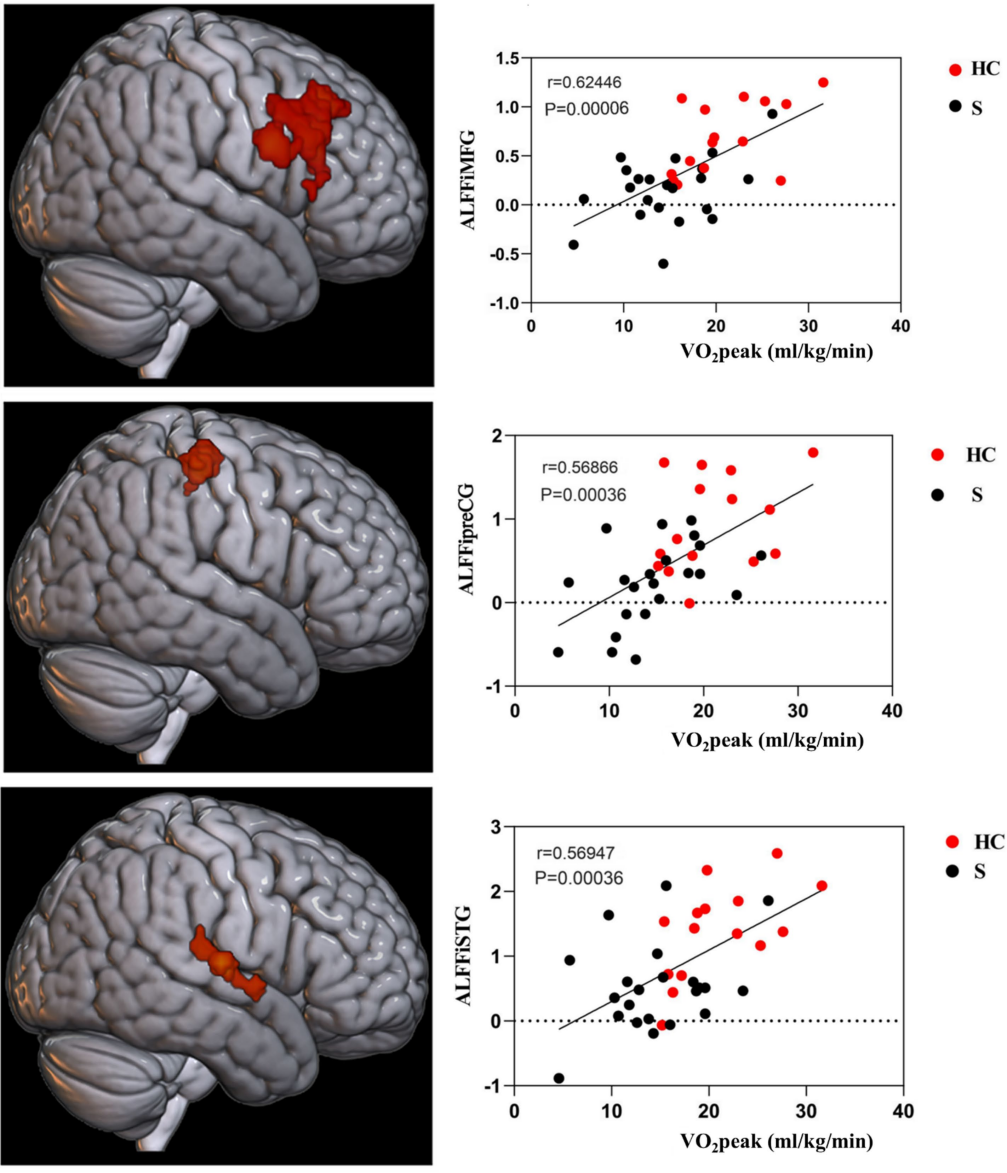
CRF-associated brain regions in stroke subjects. i, ipsilesional; MFG, middle frontal gyrus; preCG, precentral gyrus; STG, superior temporal gyrus.

The brain regions that exhibited differences in ALFF between stroke-afflicted individuals and healthy controls, and those regions demonstrating a significant convergence between the ALFF and CRF correlation analyses of the two groups were associated with local neural activity in patients with stroke. The correlation coefficients for these regions were *r* = 0.62446 (*p* = 0.00006), *r* = 0.56866 (*p* = 0.00036), and *r* = 0.56947 (*p* = 0.00036), representing the MFG, preCG, and STG, respectively, on the ipsilesional side (Figure 4).

### 3.3 FC

**Figure 5:**
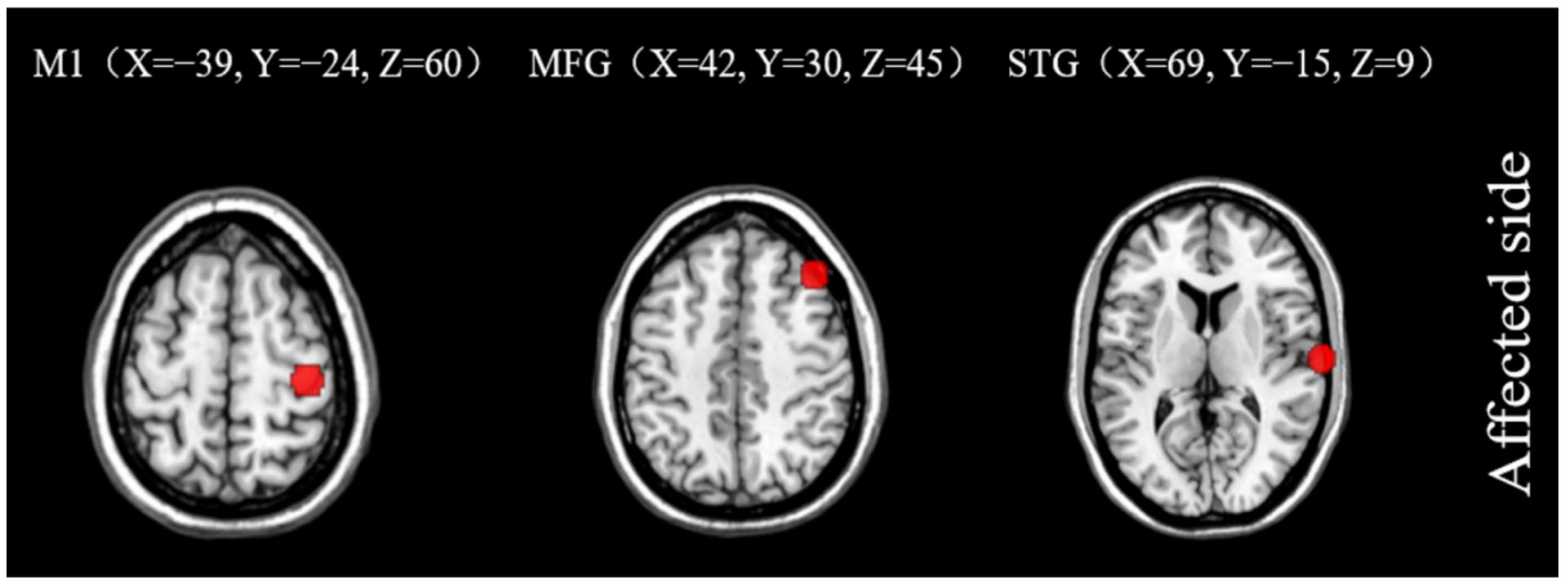
Seed points of interest coordinate diagram. M1, primary motor cortex; MFG, middle frontal gyrus; STG, superior temporal gyrus.

Subsequent to the ALFF findings, further analysis of brain networks was conducted, whereby functional connections were established based on seed points. The seed points selected for this analysis were the primary motor cortex (M1), MFG, and STG, all located on the ipsilesional side. The MNI coordinates for the center of each seed point were as follows: (*X* = −39, *Y* = −24, *Z* = 60), (*X* = 42, *Y* = 30, *Z* = 45), and (*X* = 69, *Y* = −15, *Z* = 9) for M1, MFG, and STG, respectively. The entire brain was subjected to resting-state FC analysis (Figure 5).

**Figure 6:**
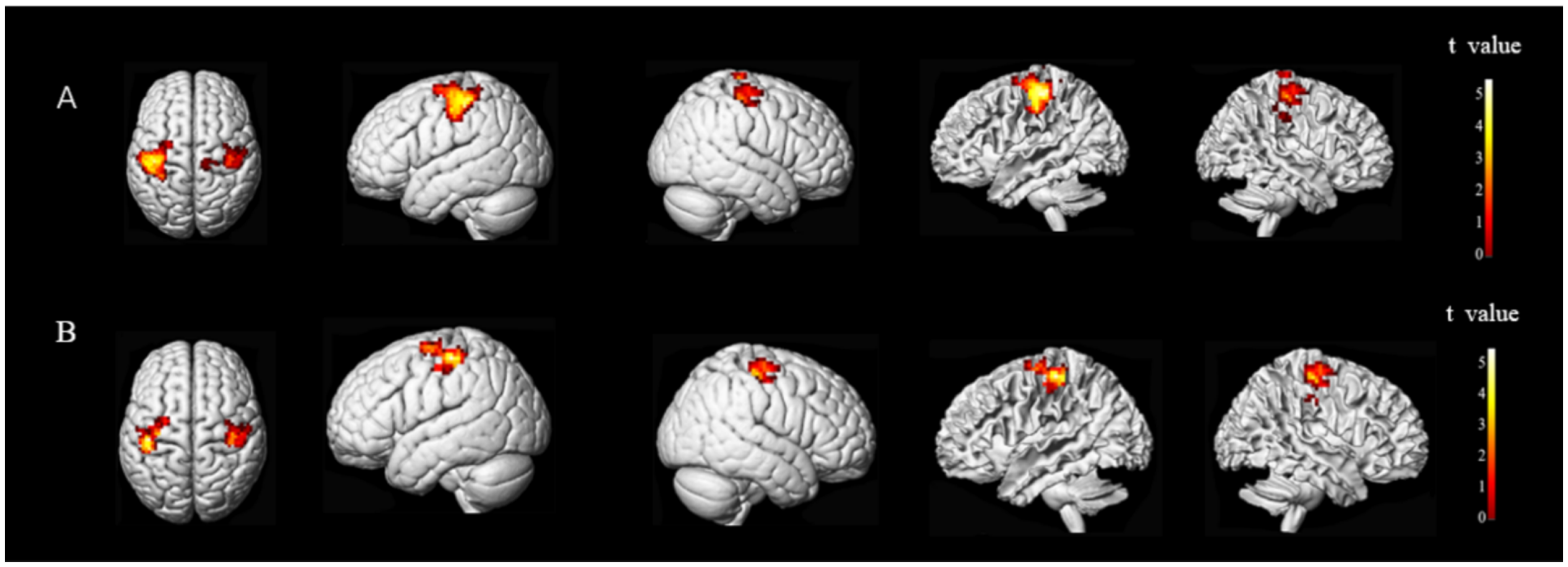
Different FC brain regions based on the M1 on the ipsilesional side of the two groups.

**Figure 7:**
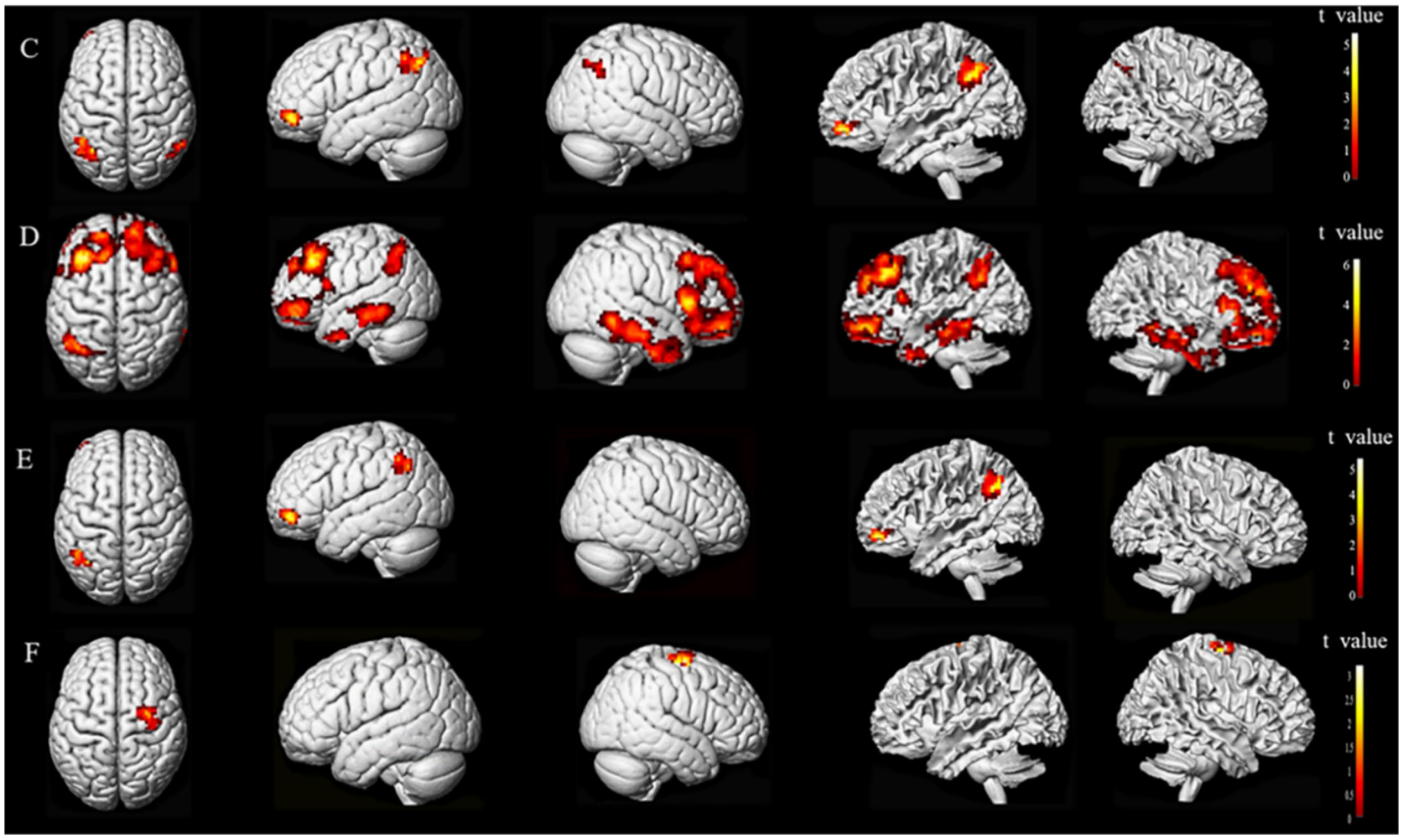
Brain regions with FC differences based on the MFG on the ipsilesional side.

The results showed a difference in the FC of stroke subjects. The overlapping brain network associated with the FC and CRF of all subjects ran from the M1 of the ipsilesional side to the preCG of the ipsilesional side and the postcentral gyrus (poCG) of the contralesional side, the MFG of the ipsilesional side to the MFG of the contralesional side, the MFG of the ipsilesional side to the Angular Gyrus (ANG) of the contralesional side, and the MFG of the ipsilesional side to the superior frontal gyrus (SFG) of the ipsilesional side. The overlapping brain region seemed to be associated with CRF in stroke subjects (Figures 6, 7).

### 3.4 Schematic diagram of FC and CRF-related brain regions in patients with stroke

**Figure 8:**
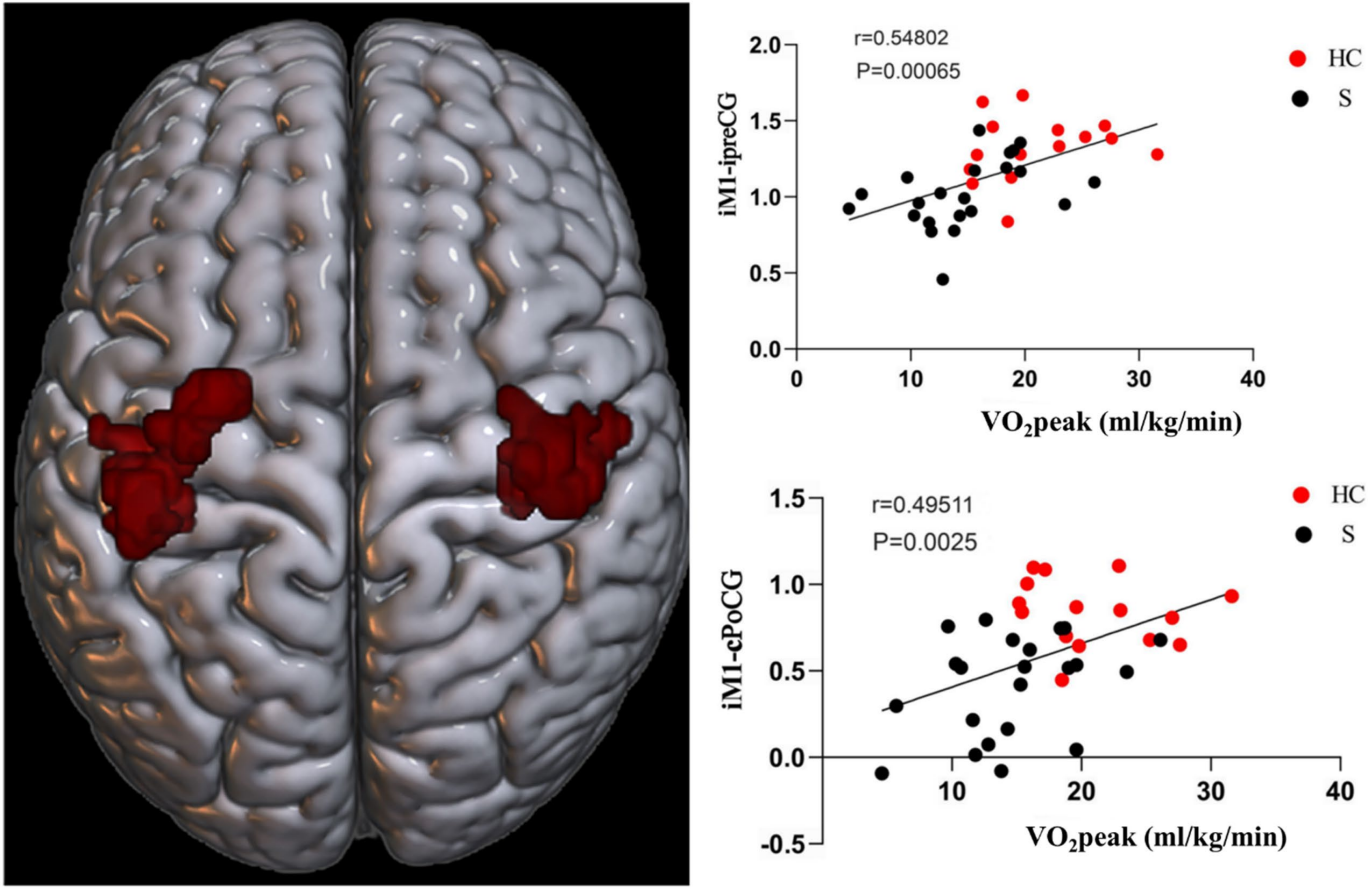
Functional connectivity (FC) of M1 on the ipsilesional side was positively correlated with CRF in stroke patients. i, ipsilesional; c, contralesional; preCG, anterior central gyrus; PoCG, postcentral gyrus.

The FC between M1 and preCG on the ipsilesional side of stroke subjects was positively correlated with CRF (*r* = 0.54802, *p* = 0.00065). The FC of M1 on the ipsilesional side and poCG on the contralesional side were also positively correlated with CRF (*r* = 0.49511, *p* = 0.0025) (Figure 8).

**Figure 9:**
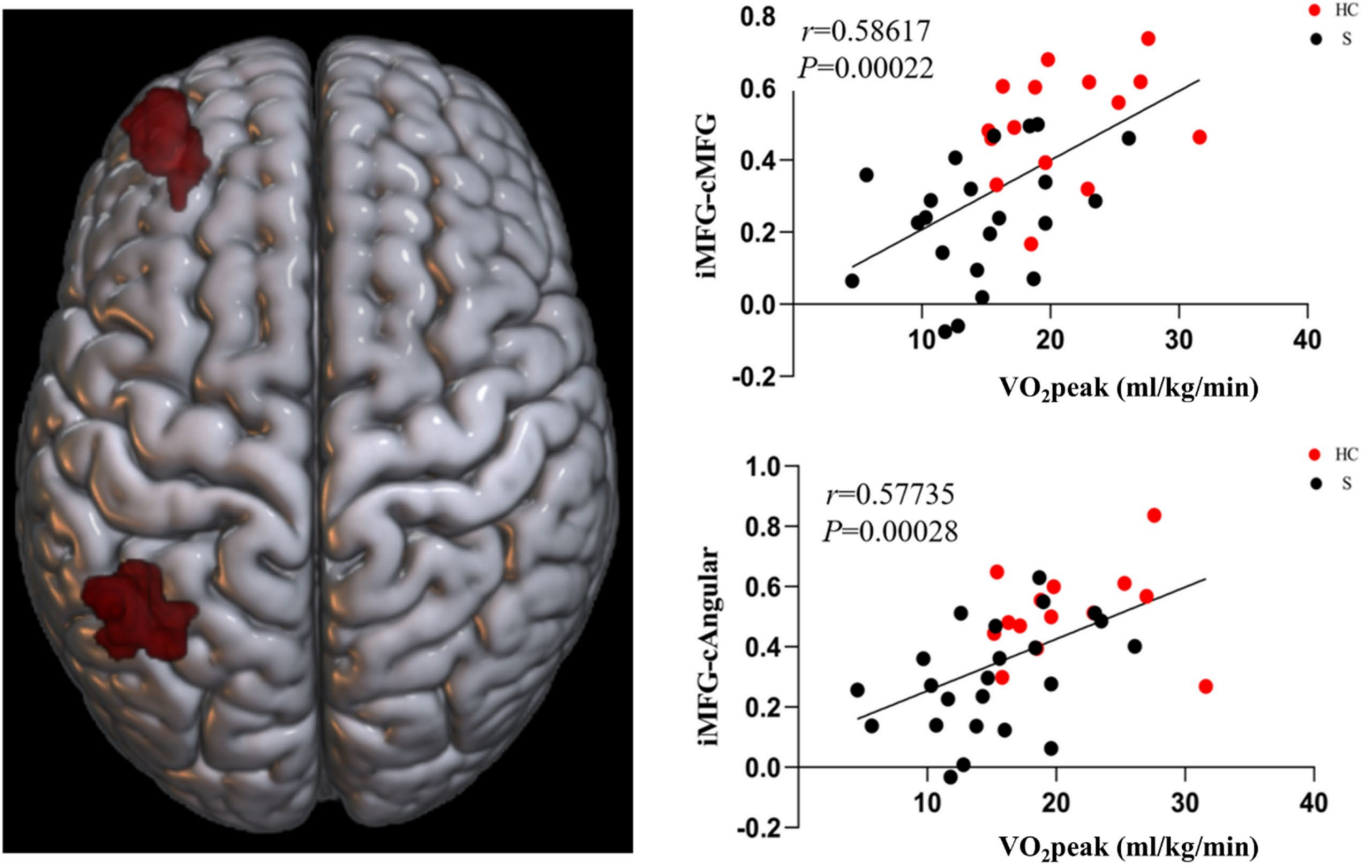
Functional connectivity of MFG on the ipsilesional side was positively correlated with CRF in stroke patients. c, contralesional; MFG, middle frontal gyrus; ANG, angular gyrus.

**Figure 10:**
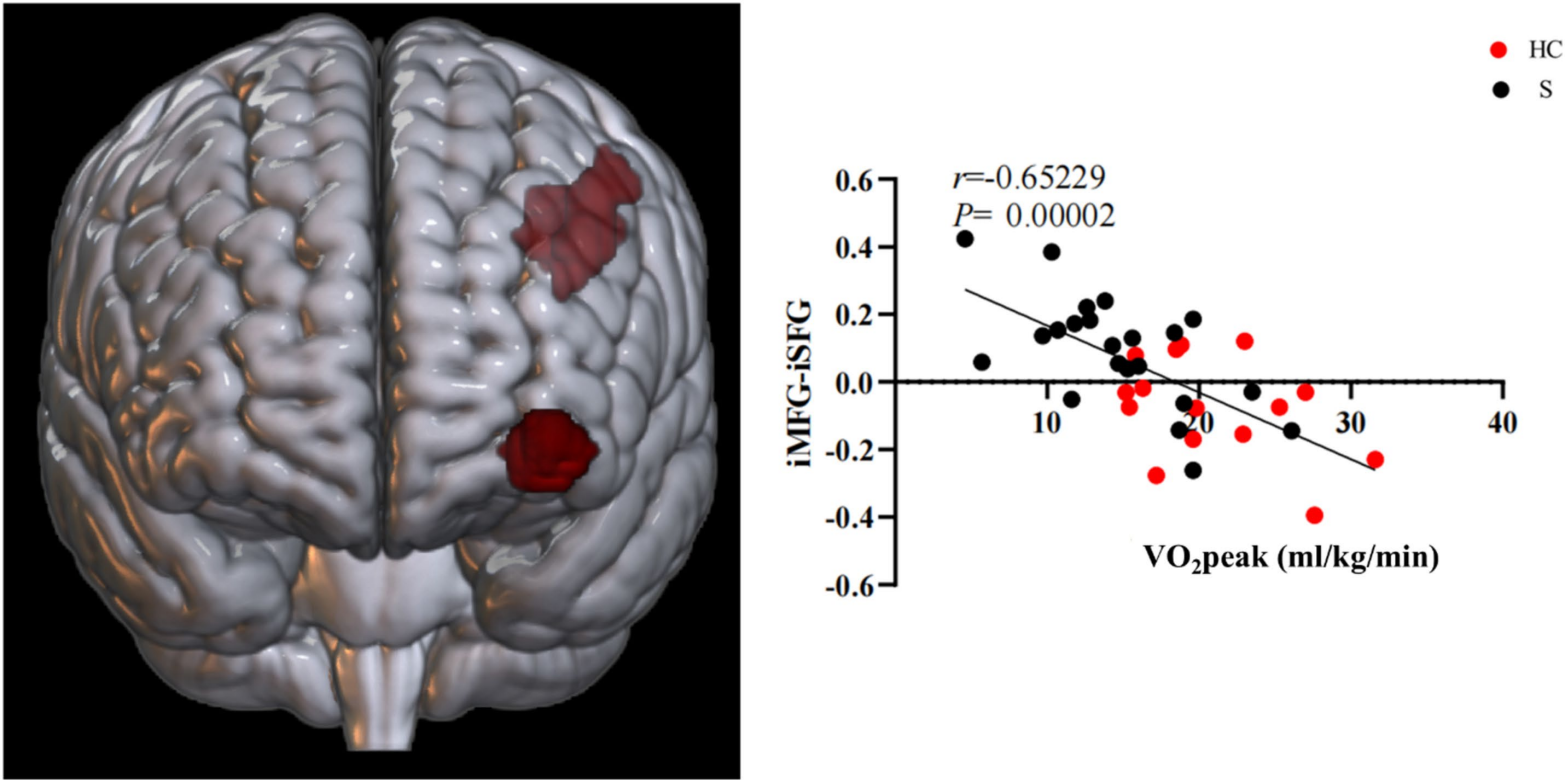
Functional connectivity of MFG on the ipsilesional side was negatively correlated with CRF in stroke patients. i, ipsilesional; MFG, middle frontal gyrus; SFG, superior frontal gyrus.

Based on the FC of the ipsilesional MFG in patients with stroke, the FC of the injured MFG and the contralesional MFG was positively correlated with CRF (*r* = 0.58617, *p* = 0.00022). Also positively correlated with CRF was the functional connection between the ipsilesional MFG and the contralesional ANG (*r* = 0.57735, *p* = 0.00028). Whereas the functional connection between the ipsilesional MFG and SFG was negatively correlated with CRF (*r* = −0.65229, *p* = 0.00002) (Figures 9, 10).

## 4 Discussion

In this cross-sectional study, rs-fMRI was used to identify potential CRF-related brain regions and networks in patients with stroke based on two levels of local neural activity. By comparing the ALFF values of healthy subjects with those of patients with stroke, we discovered increased ALFF values for the ipsilesional STG, MFG, and preCG regions in patients with stroke. Based on the ALFF analysis, brain regions related to CRF were selected as seed points for FC analysis. The results showed that the functional connection strength of stroke subjects was related to the ipsilesional preCG, contralesional poCG, contralesional MFG, contralesional ANG, and ipsilesional SFG.

First, by comparing the clinical indicators of patients with stroke and healthy subjects, we found that the 6MWD, 10mWT, CRF, and peak work-rate of patients with stroke were all far lower than those of healthy subjects, but not the FTSST, which demonstrated that stroke patients exhibit lower CRF levels compared to age-and sex-matched healthy controls. After stroke, motor function is impaired, and there is a decline in CRF closely related to motor function, which eventually results in patients being unable to carry out normally daily behavior and activities, leading to a further decline in their functional ability. CRF is one of the five vital signs (6) and plays a fundamental role in the recovery of various functions (including motor function and cognition). In this study, CPET was used to accurately measure CRF and peak work-rate, and the differences between the two groups were compared. Our results showed that the CRF of patients with stroke was reduced by about 28% compared with that of healthy people of the same age, which is consistent with previous studies (8).

Secondly, resting-state ALFF is an effective data-driven analysis technique based on fMRI (18) that effectively reflects changes in spontaneous neural activity after stroke (19). An elevated ALFF value suggests increased local neural activity. When we conducted a comparative analysis of ALFF values, we observed significantly higher ALFF values in the MFG and preCG of the ipsilesional side of healthy controls than those of patients with stroke. Conversely, the ALFF values of the bilateral SPL and MTG on the ipsilesional side were significantly higher in patients with stroke than healthy controls.

As a higher motor function center, the preCG is involved in alertness, selection, and preparation for performing tasks. The MFG is part of the prefrontal lobe, and previous studies have shown that it is closely related to higher cognitive functions such as attention, executive function, and emotion (20, 21). After stroke, the excitability of the brain hemisphere on the ipsilesional side decreases due to the death of neurons, so the neuronal activity of the MFG and preCG on the ipsilesional side decreases, resulting in a decrease in ALFF. The SPL is part of the parietal lobe and plays a key role in many cognitive, perceptual, and motor related processes (22). The SPL is a component of the default network, and changes in this network affect the motor recovery of patients with stroke (23). Given the variability in the onset of stroke in subjects in this study and the known association between brain activity of patients with stroke and their onset course, it is plausible that the elevated ALFF values observed in the bilateral SPL of patients with stroke may have been attributable to compensatory mechanisms. The MTG, being a constituent of the temporal lobe, is associated with default, motor, memory, and auditory networks. Following impairments in related functions in patients with stroke, a compensatory elevation in brain activity ensues, which may account for the augmented ALFF value observed in the affected hemisphere of the MTG.

Analysis of ALFF values and CRF values in patients with stroke showed that the injured STG, MFG, and preCG were positively correlated with CRF in patients with stroke, and it is possible that CRF is associated with local nerve activity in these related brain regions in patients with stroke. The reduction in CRF in patients with stroke across all stages of the condition has a major impact on their ability to recover motor and other functions, as well as to varying degrees on their morbidity and stroke recurrence rates. An investigation conducted by Wittfeld and his team on an elderly population in good health demonstrated that CRF was linked to the majority of cortical networks, with the most notable correlations observed in the default mode network’s prefrontal, middle temporal, and parahippocampal gyrus (24). Both the preCG and MFG are integral components of the frontal lobe and crucial constituents of the motor network. CRF and motor function are closely intertwined, which suggests there is an association. Despite the subjects in this study not being entirely congruent with those in Wittfeld et al.’ (24) study, both studies have preliminarily substantiated that there is an intimate relationship between the frontal lobe and CRF. Hence, our conjecture posits that the frontal lobe has significance in relation to CRF. The STG, as a crucial component of the default network, may assume a relatively prominent function in the CRF of individuals with stroke. Nevertheless, the current dearth of pertinent research necessitates further empirical medical evidence to elucidate the mechanism by which the STG influences CRF.

Functional connectivity is a widely used method for examining brain network activity, with the strength of FC serving as an indicator of the level of connectivity between different regions of the brain. Based on the findings from the ALFF analysis, the ipsilesional STG, MFG, and primary motor cortex (M1) were identified as seed points for conducting a comprehensive FC analysis of patients with stroke, with the aim of identifying brain regions associated with CRF and brain network activity. FC analysis using the seed point of the ipsilesional STG revealed no discernible distinction between the two groups. However, when the analysis was conducted using the ipsilesional MFG as the seed point, the functional connection between the bilateral MFG and the ipsilesional ANG was observed to be significantly weakened. Conversely, the functional connection with the ipsilesional MFG was found to be enhanced. Patients with stroke exhibited a significant reduction in functional connection strength between ipsilesional M1 and ipsilesional preCG as well as contralesional poCG, as determined by their functional connectivity. The functional connections associated with CRF in patients with stroke were identified as contralesional MFG, ANG, and SFG, with the former two displaying a positive correlation with CRF, and the latter exhibiting a negative correlation. Furthermore, functional connections between ipsilesional preCG and contralesional poCG were positively correlated with CRF, as determined by their functional connectivity with ipsilesional M1.

The MFG is part of the motor network and plays an important role in attention control (25). The ANG, within the Inferior Parietal Lobule (IPL), is an important component of the frontal parietal motor network (26). In the present study, we observed a reduction in the functional connection strength of the ipsilesional MFG, contralesional MFG, and ANG, indicating a potential association between CRF and the motor network in individuals with stroke. Additionally, the functional connection strength of the MFG and SFG in the ipsilesional hemisphere was found to be augmented in patients with stroke, and it exhibited a negative correlation with CRF. One possible explanation is that the injury resulted in a decrease in the excitability of the cerebral hemispheres, leading to compensatory mechanisms being activated to sustain brain function. This over-compensation may disrupt functional equilibrium and the coherence between the hemispheres, which are fundamental for maintaining functional stability (26), and therefore, lead to a decline in CRF. M1 is a prominent brain region implicated in the motor network. Research has demonstrated a noteworthy association between a reduction in the strength of interhemispheric motor network functional connectivity and motor function recovery outcomes. Conversely, no correlation has been found between intra-hemispheric functional connectivity and motor function recovery outcomes (27). In this study, the functional connectivity strength of the motor network in the hemispheres of patients with stroke was positively correlated with CRF, and clinical observations indicate that motor function in patients with stroke is closely correlated with CRF. This suggests that the brain regions related to CRF in patients with stroke are similar and different from the brain regions of the motor network, which indirectly indicates that motor function is not the only factor affecting CRF. The functional connectivity strength of ipsilesional M1 and contralesional poCG was positively correlated with CRF. M1 and poCG are core components of the sensorimotor network and play crucial roles in human movement. Previous studies on motor function recovery after stroke found that the functional connection strength of ipsilesional M1 and the sensorimotor network on the contralesional side decrease after patients with stroke (28), and motor function is closely related to CRF; therefore, this also may be why the functional connection strength of ipsilesional M1 and contralesional poCG is positively correlated with CRF.

In general, we observed lower CRF levels in stroke patients compared to healthy individuals. However, our cross-sectional study design limits our ability to determine if the reduced CRF is a direct consequence of stroke or predated the event. Physical inactivity is known to increase stroke risk, suggesting that differences in CRF levels may have existed before the stroke. Therefore, we cannot exclude the influence of pre-stroke physical activity on post-stroke CRF. These findings highlight the need for longitudinal studies to better understand the causal relationships between physical activity, stroke, and CRF. Furthermore, most of the brain regions that correlated with CRF in patients with stroke, such as the MFG, preCG, and poCG, are components of the sensorimotor network. An important reason for this may be that motor function is closely related to CRF, and sensory function affects the recovery of motor function. Various constituent brain regions of the default mode network (DMN) are also related to CRF, such as the ANG and STG. The basic function of the DMN is in maintaining the functions of other networks. Therefore, how it plays a role in brain networks related to CRF needs to be further dissected.

### 4.1 Conclusion

The CRF, peak work-rate, 10mWT and 6MWD of stroke subjects were lower than those of healthy controls, while the results for the FTSST were the opposite. Our rs-fMRI index ALFF analysis highlighted several brain regions and areas of local neural activity that appeared to be related to CRF in patients with stroke: the ipsilesional STG, MFG, and preCG. Additionally, FC showed several brain regions and networks were correlated with CRF in stroke patients, i.e., the ipsilesional M1 to ipsilesional preCG and contralesional postcentral gyrus, ipsilesional MFG to contralesional MFG, ANG and ipsilesional SFG.

## Data Availability

The original contributions presented in the study are included in the article/Supplementary material, further inquiries can be directed to the corresponding author.
