# Supplementary material for: A resting-state fMRI cross-sectional study of cardiorespiratory fitness decline after stroke
Source: Front Neurol. 2025 Jan 28;16:1465467. doi: 10.3389/fneur.2025.1465467 (PMC11877007; doi:10.3389/fneur.2025.1465467)
Supplement: Supplementary file 1 [file Presentation_1.pdf]

Supplementary material

MRI Data Acquisition

MRI data for this study were acquired at Huashan Hospital, Fudan University, using a 3.0T GE MR750 MRI scanner. During the scanning procedure, participants were instructed to remain motionless, and sponge cushions were utilized to stabilize the head. To minimize psychological and emotional distress caused by scanner noise, participants were provided with soft earplugs. A physician was present throughout the data acquisition process to monitor for any adverse events. In the event of participant discomfort, scanning was immediately terminated, and appropriate symptomatic treatment was administered by the attending physician.

The MRI protocol included the following sequences:

T1-weighted imaging: Axial T1-weighted FSPGR sequence for initial localization (sagittal plane, 192 slices, slice thickness 0.9 mm, no interslice gap, TR = 1400 ms, TE = 3.22 ms, FOV = 240 mm × 240 mm, flip angle = 12°, acquisition matrix 256 × 256).

T2-weighted imaging: Axial T2-weighted GRE sequence (20 axial slices, slice thickness 10 mm, no interslice gap, TR = 5000 ms, TE = 120 ms, FOV = 220 mm × 220 mm, acquisition matrix 64 × 64).

Resting-state fMRI (rs-fMRI): Echo-planar imaging (EPI) sequence (43 axial slices, slice thickness 3 mm, no interslice gap, TR = 2000 ms, TE = 30 ms, FOV = 220 mm × 220 mm, acquisition matrix 64 × 64, flip angle = 90°, total acquisition time = 8 minutes, 240 time points collected).
